# Comparison of three image segmentation techniques for target volume delineation in positron emission tomography

**DOI:** 10.1120/jacmp.v8i2.2367

**Published:** 2007-03-09

**Authors:** Laura Drever, Wilson Roa, Alexander McEwan, Don Robinson

**Affiliations:** ^1^ Department of Medical Physics BC Cancer Agency Victoria British Columbia Canada; ^2^ Department of Radiation Oncology Cross Cancer Institute Edmonton Alberta Canada; ^3^ Department of Oncologic Imaging Cross Cancer Institute Edmonton Alberta Canada; ^4^ Department of Medical Physics Cross Cancer Institute Edmonton Alberta Canada

**Keywords:** PET, target volume delineation, threshold segmentation, Sobel segmentation, watershed segmentation

## Abstract

Incorporation of positron emission tomography (PET) data into radiotherapy planning is currently under investigation for numerous sites including lung, brain, head and neck, breast, and prostate. Accurate tumor‐volume quantification is essential to the proper utilization of the unique information provided by PET. Unfortunately, target delineation within PET currently remains a largely unaddressed problem. We therefore examined the ability of three segmentation methods—thresholding, Sobel edge detection, and the watershed approach—to yield accurate delineation of PET target cross‐sections. A phantom study employing well‐defined cylindrical and spherical volumes and activity distributions provided an opportunity to assess the relative efficacy with which the three approaches could yield accurate target delineation in PET. Results revealed that threshold segmentation can accurately delineate target cross‐sections, but that the Sobel and watershed techniques both consistently fail to correctly identify the size of experimental volumes. The usefulness of threshold‐based segmentation is limited, however, by the dependence of the correct threshold (that which returns the correct area at each image slice) on target size.

PACS numbers: 87.58.Fg, 87.57.Nk

## I. INTRODUCTION

The goal of modern radiotherapy is to achieve improvements in local tumor control through dose escalation to diseased tissue and, at the same time, to minimize the damage to surrounding normal structures. Powerful new delivery techniques such as dynamic multileaf collimation and helical tomotherapy have the potential to conform delivered dose to well‐defined volumes with unprecedented precision; however, accurate target identification and delineation pose limiting factors.

Due to high spatial resolution and fidelity, images provided by X‐ray computed tomography (CT) serve as the current basis for planning radiation therapy treatments. Although the electron densities provided by CT are essential to modern heterogeneity‐based dose calculation algorithms, interest in augmenting CT with information gained from other imaging modalities is great.^(^ ^1^ ^,^ ^2^ ^)^ Positron emission tomography (PET) as an adjuvant image modality in radiation therapy is attracting much of this interest because of its ability to provide valuable physiologic data. Utilization of such data promises to greatly aid in the accurate delineation of tumor volumes through the identification of biologic activity. For example, PET has been shown to have greater sensitivity and specificity than either CT or magnetic resonance imaging in the staging of lung cancer.^(^ ^3^ ^–^ ^5^ ^)^ The delineation of lung‐tumor boundaries in CT images can be complicated by the presence of atelectasis, pleural effusion, pneumonitis, and displacement of normal tissue. Lymph node involvement, which is always of concern, is difficult to distinguish using CT scans alone. Functional information gained from PET imaging may help to overcome those limitations. The incorporation of PET information into radiotherapy planning is also currently under investigation in numerous other sites, including brain,^(6)^ head and neck,^(7)^ breast,^(8)^ and prostate.^(9)^

A key step to proper utilization of the unique information provided by PET is accurate tumor volume quantification. Unfortunately, target delineation within PET currently remains a largely unresolved problem.^(10)^ To date, the delineation of PET images has relied upon either the judgment of a nuclear medicine physician or threshold segmentation based on standard uptake values^(^ ^10^ ^–^ ^15^ ^)^ or local contrast.^(^ ^16^ ^,^ ^17^ ^)^ As evidenced by the literature, no universal agreement currently exists concerning the appropriate threshold level (or range of levels) required to accurately outline PET target volumes.

Investigations of analytic PET segmentation schemes apart from threshold techniques have yet to appear. A number of factors, including low resolution (~5 mm), image noise, and spatial aberrations arising from detector geometry and data processing contribute to the difficulties in interpreting PET data so as to accurately delineate volume. Identification of the precise geometric boundary separating tumor from its surrounding background can, in many cases, prove vexing.

The current difficulties associated with accurate geometric interpretation of PET data pose fundamental limitations to the full utilization of this powerful imaging modality for planning more accurate and effective radiotherapy treatments. If the full potential of PET is to be properly assessed and utilized, improvement in this situation is an immediate need. We therefore examined three segmentation methods for their ability to yield accurate delineation of PET target volumes: a local contrast‐based threshold technique, Sobel edge detection, and a watershed approach. The local contrast‐based threshold technique is relatively simple; Sobel edge detection is slightly more sophisticated; and the watershed approach is relatively mathematically complex. A phantom study employing well‐defined volumes and activity distributions provided an opportunity to assess the relative efficacy of these three approaches to yield accurate target delineation in PET.

The threshold technique presented here differs from those previously used^(^ ^16^ ^,^ ^18^ ^)^ in that it focuses on the delineation of individual cross‐sections rather than just on total volume.

## II. THEORY

Segmentation poses one of the most challenging problems in image processing today. Methods abound, and their characteristics can vary significantly according to the specific application and imaging modality. Currently, no single existing segmentation scheme will yield acceptable results over the entire spectrum of medical image types that may be contemplated.

Classic image segmentation may be defined as the partitioning of an image *I* into distinct constituent subregions or subsets, $S_{i}$, which are themselves homogeneous with respect to some defining characteristic. Thus, these subsets must satisfy the relations

(1) $$\begin{array}{llll} S_{i}\subset I & I=\overset{N}{\underset{i=1}{\cup S_{i}}} & S_{i}\cap S_{j}=Ø & \forall i\neq j\;\;, \end{array}$$

and every $S_{i}$ is connected. When the constraint of connectedness is relaxed, as is often the case in medical image segmentation, the subsets $S_{i}$ are called “classes.” A further relaxation may be instituted by removing the constraint of non‐intersection between subsets so as to encompass partial‐volume effects in which a single image element (pixel) may belong to multiple tissues. The present investigation retains the classic definition of segmentation.

### A. Thresholding

Thresholding is a simple and intuitive method of segmentation in which all pixels that meet a given criteria are regarded as belonging to the target, while all others are relegated to background status. For medical applications, the challenge is to determine a partition value τ such that the target image corresponds to a meaningful clinical entity.

### B. Sobel edge detection

Sobel edge detection determines segmentation according to maximums in the absolute value of the gradient of the image. It has the advantage over most other edge detection methods of being less sensitive to image noise, and it can also yield estimates of edge direction and magnitude, which can be useful for subsequent processing. Of course, digital images present intensity as a discrete, rather than continuous, function of spatial location and cannot be differentiated analytically; hence, numerical approximations to the gradient must be employed. These numerical approximations are often represented as 3×3 matrices that operate on the image and are called “masks.” Atypical mask structure,

(2) $$\left[\begin{array}{lll} w_{1} & w_{2} & w_{3}\\ w_{4} & w_{5} & w_{6}\\ w_{7} & w_{8} & w_{9} \end{array}\right]\;\;,$$

consists of matrix elements, each with a weight value to be applied in the calculations. The response of the mask is defined at the center of the matrix,

(3) $$R=w_{1}I_{1}+w_{2}I_{2}+\ldots +w_{9}I_{9\;\;,}$$

where $I_{i}$ is the gray level of the pixel being operated on, and $w_{i}$ is a mask coefficient.

After application of the mask, the remaining steps in numeric edge detection are identical to those in analytical edge detection.

### C. Watershed

Based on concepts from mathematical morphology, watershed segmentation is a hybrid technique that combines edge detection and region identification. Unlike the Sobel and threshold approaches, the watershed approach yields closed contours.

The watershed method treats the gradient of the image as topography in which greater pixel values correspond to higher points in the landscape. Conceptually, this method searches for points in the topography at which a drop of water is equally likely to run off in more than one direction. Connecting those points creates the “watershed lines” that segment the image. Another way to visualize the technique is to imagine that wells have been drilled at all the local minima and that the water table begins to rise. Eventually, water from one local minimum joins with water from a second local minimum; dams are built to prevent the water from joining. In the end, all that is left of the landscape is the dams—the watershed segmentation lines. Gonzalez and Woods have discussed the mathematical details of this segmentation technique in detail.^(19)^

## III. METHODS AND MATERIALS

A phantom study employing simple geometric objects was used to investigate the relative efficacy of the threshold, Sobel, and watershed segmentation approaches to accurately delineate F18‐activated volumes in PET. Experimentally derived cross‐sectional areas and volumes were then rigorously compared with their physical (“true”) values. A phantom study eliminates the uncertainties associated with an assessment based on clinical data, which, of necessity, involves uncertainties in both the physical extent of the tumor and the activity distributions encountered.

Hollow cylinders and spheres constructed of polymethyl methacrylate (PMMA) served as target volumes. These were placed within a larger PMMA cylinder, which itself could be loaded with activity to provide a surrounding background.

The cylinders, whose axis of rotation was placed orthogonal to the image plane and coincident with that of the external cylindrical phantom, provided volumes with cross‐sectional areas that were, apart from end‐effects, invariant in the longitudinal dimension. They yielded data in which partial‐volume effects were restricted to the two dimensions of the image plane. In contrast, the spheres exhibited cross‐sectional variations in all three dimensions (in closer accord with actual clinical volumes).

Cylinders were constructed with inside diameters of 12.4 mm, 25.4 mm, and 47.5 mm, and a wall thickness of 4 mm. Spheres were fabricated with inside diameters of 23 mm, 35 mm, and 59 mm, and a wall thickness of 1 mm. These diameters were all chosen to represent the range of small to large tumors observed clinically.

**Figure 1:**
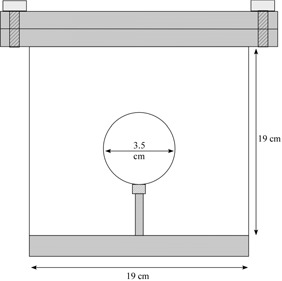
Sectional diagram of the medium‐diameter spherical target within the surrounding background cylinder.

For imaging, the cylinders and spheres were suspended by a PMMA support rod within the larger outer cylindrical phantom (Fig. 1). Internal and external volumes were both filled with F18 in water suspension. Activity concentrations used for background and target volumes ranged from 1.5 kBq/mL to 14.1 kBq/mL and 7.7 kBq/mL to 78.5 kBq/mL respectively. These loadings resulted in activity concentration ratios between the volumes of the internal target and the external surrounding background ranging from approximately 2:1 to 15:1 [target‐to‐background activity concentration (TBAC) ratio] in accord with values found in the literature^(^ ^18^ ^,^ ^20^ ^)^ and seen clinically.

Optimum image quality is, in general, associated with peak noise equivalent count (NEC) rates. Published peaks (which are fairly broad) of the NEC curve for the CPET Plus scanner (Philips Medical Systems, Andover, MA) used in the present work are associated with activity concentrations that range from 3.8 kBq/mL to 12.3 kBq/mL, depending on the particular technique used.^(21)^ Activity concentrations used to create the background, which constituted more than 95% of the total volume imaged, were chosen in accordance with that range. According to NU 2–2001 analysis, sensitivity for the CPET Plus is 3.0 cps/kBq, and the scatter fraction is 35%. The peak NEC rate is 14 kcps at an activity of 3.8 kBq/mL.

All measurements were conducted with the axis of rotation of the external cylinder orthogonal to the image plane and coincident with the center of reconstruction. All internal target volumes were radially centered within the external cylinder. These internal volumes were also centered within, and fully encompassed by, the axial field of view. The phantom was scanned with a CPET Plus scanner using a clinical whole‐body protocol, with a slice width of 4 mm and in‐plane voxel dimensions of $3\times 3\;{\text{mm}}^{2}$. To maintain a constant cumulative activity within the target, acquisition durations were adjusted to account for decay. Data sets for the cylindrical targets were acquired in 7–20 minutes, and for the spherical targets, in 6–15 minutes. All images acquired for the study were reconstructed using the same ^137^Cs attenuation‐corrected three‐dimensional row action maximum likelihood algorithm (3D RAMLA) employed clinically.^(^ ^22^ ^,^ ^23^ ^)^ The exact details of the implementation of this algorithm are withheld as proprietary by the vendor.

The CPET Plus scanner has a 25.6‐cm axial field of view and a detector ring of 90 cm diameter composed of six curved sodium iodide [NaI(Tl)] crystals that measure 47 cm circumferentially, 30 cm axially, and 2.54 cm in thickness. Each crystal is sampled by 48 photomultiplier tubes, each with a diameter of 6.2 cm. Coincidences are registered between opposing detectors (“on” pairs) and the two detectors adjacent to the opposing detectors (“off” pairs). All images are acquired in 3D mode (no septa) and internally binned in 2×2×2‐mm voxels. Spatial resolution for a point source in the transaxial direction is reported to be 4.6 mm (full width at half maximum) at the center, and the axial resolution is 5.7 mm.^(21)^

The PET data are acquired in a volumetric manner, spanning a length of 256 mm in the axial direction. After processing, data are presented to the user as a series of images that may include axial, coronal, and sagittal views. For the present work, the axial images alone were analyzed, because these are the common format in which CT data are compared and augmented.

To ensure uniform activity distributions within the target and the surrounding background volumes, great care was taken to ensure thorough mixing of F18 within the water suspension. All images were analyzed using a MATLAB (MathWorks, Natick, MA) program developed in‐house. For the cylinders and the spheres, we compared cross‐sections determined by each of the three segmentation approaches to their true physical values as a function of TBAC ratio and target cross‐section size. For the threshold technique, the variability with respect to percentage contrast level was also investigated. Agreement was deemed to occur when cross‐sectional areas differed by less than 5%, and centroids deviated by less than 2 mm. For the cylindrical and spherical targets alike, all three methods were analyzed slice‐by‐slice. Each image in the data set was treated independently; no pixel connectivity constraints were imposed across slices. This method of segmenting the PET images was chosen because it approximates the fashion in which CT images are handled in radiation therapy treatment planning systems.

Because the slice thickness of the PET scan represents a significant fraction of the size of the spherical targets examined, the variation in target cross‐section captured in a single image cannot be safely ignored. The physical area of the sphere represented on any image cannot simply be taken as the area that occurs at either end or in the geometric middle of the slice. Rather, it must be determined as a weighted mean over the full thickness of the slice. The weighted mean target area captured on any slice was calculated using the formula

(4) $$\bar{A(x)}=\frac{\pi \int_{x-\delta }^{x+\delta }\left(r^{2}-x\prime^{2}\right)dx\prime }{\int_{x-\delta }^{x+\delta }dx\prime }=\pi \left[r^{2}-x^{2}-\frac{\delta^{2}}{3}\right]\;\;,$$

where *r* is the radius of the sphere, *x* is the distance from the center of the sphere to the center of the slice, and δ is half the slice thickness.

### A. Thresholding

Partition values τ for segmentation were derived with respect to the local contrast range at each slice location. That range is defined as the difference between the maximum pixel intensity ${\text{IT}}_{\max}$ within the target, and the effective mean pixel intensity ${\text{IB}}_{\text{mean}}$ of the background region containing the phantom, but excluding the target. The ${\text{IB}}_{\text{mean}}$ is determined by examining the image histogram.

**Figure 2:**
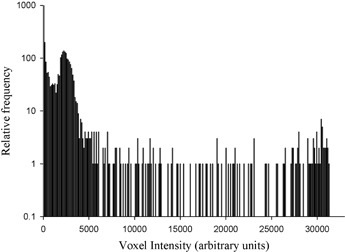
Distribution of pixel intensity values for the PET image of the 47.5‐mm–diameter cylindrical target with 10:1 target‐to‐background activity concentration ratio. The large peak at zero pixel intensity corresponds to the large number of pixels outside the phantom (null corners of the image matrix). The second large peak is centered on a pixel value of 3337; it is taken to be the mean pixel value of the background within the phantom.

Fig. 2 shows the frequency distribution of pixel values present in the PET data at the central slice location for the large 47.5‐mm–diameter target cylinder with a TBAC ratio of 10:1. The large peak at zero pixel value corresponds to the empty space surrounding the phantom and the null corners of the image matrix which result from the circular reconstruction being mapped onto a square image matrix. The second prominent peak centered at a pixel value of about 3337 corresponds to the modal pixel value within the phantom, and it is chosen to be the ${\text{IB}}_{\text{mean}}$. The large gap between the ${\text{IB}}_{\text{mean}}$ and the ${\text{IT}}_{\max}$ serves to define the contrast range between the background and target volumes. A fraction or percentage of the contrast range (% contrast level) is then used to define the partition value τ at which to perform image segmentation, according to the relationship

(5) $$\text{threshold}\;\text{intensity}\;\tau =\left(\frac{\%\;\text{contrast}\;\text{level}}{100}\right)({\text{IT}}_{\text{max}}-{\text{IB}}_{\text{mean}})+{\text{IB}}_{\text{mean}}\cdot$$

Target cross‐sections were thus determined using a simple threshold technique in which all pixels with a value greater than that corresponding to the percentage contrast level were deemed to belong to the volume of interest; all others were designated as background.

### B. Sobel edge detection

The absolute value of the image gradient |∇I(x,y)| is numerically determined for each pixel in the image. If the absolute value of the gradient at a specific location is greater than some threshold value (12% of the maximum pixel per frame for the cylinders, and 12% of the maximum pixel of the entire image set for the spheres), then that point is designated a candidate edge point. (These lower bounding threshold values were chosen to prevent detection of edges that result from image reconstruction artifacts within the surrounding background volume.)

This method yields overly thick edges that are thinned by comparing the horizontal and vertical partial derivative at each candidate edge point. If the partial derivative is maximum in the horizontal and vertical directions alike, then the candidate edge point is determined to be an edge point. However, if a maximum exists in only one direction, then the point is designated to belong to an edge only if its magnitude is twice that of the gradient in the other direction.^(24)^ The gray‐scale PET images are analyzed using the Sobel masks

(6) $$\left[\begin{array}{ccc} -1 & 0 & 1\\ -2 & 0 & 2\\ -1 & 0 & 1 \end{array}\right]\;\;\text{and}\;\;\left[\begin{array}{ccc} \;\;1 & \;\;2 & \;\;1\\ \;\;0 & \;\;0 & \;\;0\\ -1 & -2 & -1 \end{array}\right]\;\;,$$

which yield horizontal and vertical edges respectively.

### C. Watershed

The watershed technique was applied to the gradient vector flow (GVF)^(25)^ of each image rather than to their respective gradients. Use of the GVF results in images in which rapid change in pixel intensity occurs near edges, thus providing for a more robust analysis.^(26)^ To allow for more efficient processing, the GVF of each image is calculated and mapped onto 256 gray levels.

The problem of over‐segmentation, which often results from simple implementation of the watershed technique, can be avoided by the judicious use of markers to limit the range over which the algorithm can operate. For the present work, two sets of markers were used, the first of which identified the inner region of the target, and the second, a local boundary external to the region of interest.^(^ ^26^ ^,^ ^19^ ^,^ ^27^ ^)^

Because the results of watershed segmentation are sensitive to marker choice, an automated method for marker identification was implemented. Marker sets were found through analysis of the images of the original PET data and of their respective GVF counterparts. The location of maximum pixel intensity in each PET image was found. Use of only a single target volume at any given time and of TBAC ratios greater than 1 guarantee that the maximum pixel intensity locations always lie within the target volume. Next, the GVF pixel intensity at the same location was determined and used to threshold the GVF image. Thresholding was performed so that all pixels with intensity values between the GVF image minimum and the threshold level were included in the region of interest.

Because pixel intensity values in the GVF image are largest near edge locations and lowest in non‐edge regions, the foregoing form of thresholding yields two disjoint regions of interest. One of these regions resides within the target volume; the other is located external to it. The watershed algorithm is then applied to the portion of the GVF image that lies between the two regions.

## IV. RESULTS

### A. Cylinders

#### *A.1 Thresholding*

**Figure 3:**
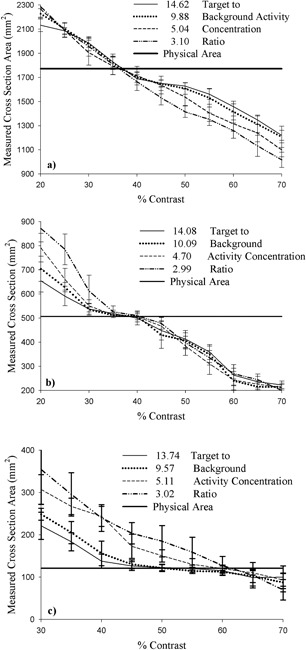
Cross‐sectional area delineated by the threshold method as a function of percentage contrast for the (a) large (47.5 mm), (b) medium (25.4 mm), and (c) small (12.4 mm) cylindrical targets for various target‐to‐background activity concentration ratios.

For the three cylinders, Fig. 3 presents experimental cross‐sectional areas derived by threshold segmentation as a function of percentage contrast level over the range of investigated TBAC ratios (from about 3:1 to approximately 15:1). As can be seen, measured area varies as a function of both percentage contrast level and TBAC ratio. The variation with activity concentration ratio is least for the largest cylinder and greatest for the smallest cylinder. For the smallest cylinder, the percentage contrast levels that yield correct area, as indicated by the horizontal line (falling within experimental error), vary from about 40% to approximately 65%. For the medium‐size cylinder, percentage contrast levels that correspond to correct area determination range from about 30% to 40%. For the largest cylinder, the percentage contrast level interval that yields correct physical area narrows to approximately 34%−40%.

#### *A.2 Sobel edge detection*

**Figure 4:**
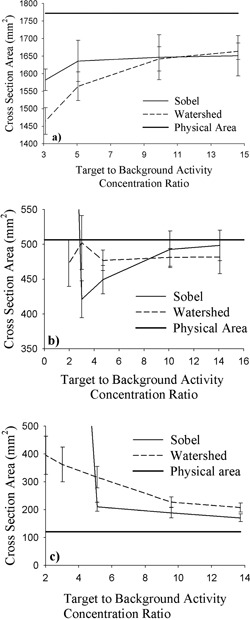
Cross‐sectional area delineated by the Sobel and watershed methods as a function of target‐to‐background activity concentration ratio for the (a) large (47.5 mm), (b) medium (25.4 mm), and (c) small (12.4 mm) cylindrical targets. The solid horizontal line corresponds to the physical area of each cylinder.

Experimental results obtained with the Sobel technique (Fig. 4) yield a decidedly poor representation of physical reality. Agreement with true physical area (within experimental error) occurs at no TBAC ratio for either the small or the large cylinder. For the small cylinder, all measured areas exceed their true size, and for the large cylinder, the opposite case holds. At TBAC ratios of 2.02 and 3.02, the Sobel method fails completely to identify the small target cylinder, delineating the external background cylinder instead. The area of the background cylinder is greater than 28,000 mm^2^, and as a result, the data points for these two TBAC ratios are well off the plot in Fig. 4(c).

Only slightly better results are obtained with the medium cylinder, with two TBAC ratios (10.0 and 14.1) yielding agreement between measurement and reality within experimental error. For the medium cylinder, measurements at TBAC ratios of 3.02 and 5.11 lie below the physical values, and at the lowest TBAC ratio (1.96), the external background cylinder is once again erroneously identified.

#### *A.3 Watershed*

Results obtained with the watershed technique (Fig. 4) prove equally disappointing. Once again, no agreement between experiment and reality is achieved for either the large or the small cylinder. As with the Sobel technique, measured values are too small for the large cylinder and too large for the small cylinder. For the medium cylinder, results reveal two TBAC ratios at which measurement agrees with physical reality, but one does so only at the very limit of experimental error. All measurements of the medium cylinder yield areas that lie below their physical value (similar to the situation with the large cylinder).

### B. Spheres

Spherical volumes present variable cross‐sectional areas as a function of slice location, and hence they present more realistic geometries that better approximate geometries associated with real tumors. As such, and in comparison with the cylindrical volumes already examined, they pose a greater challenge for accurate contour delineation.

#### *B.1 Thresholding*

**Figure 5:**
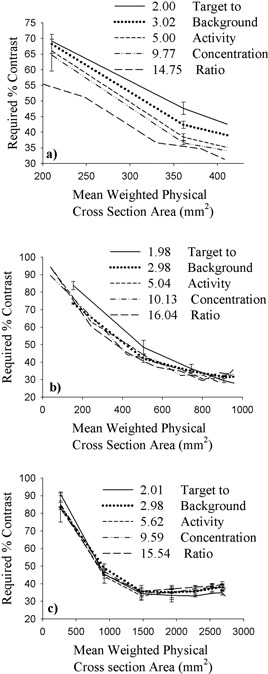
Required percentage contrast as a function of mean weighted physical cross‐section area for the (a) small‐ (23‐mm), (b) medium‐ (35‐mm), and (c) large‐diameter (59‐mm) spherical targets for various target‐to‐background activity concentration ratios.

Results obtained with the cylinders clearly indicate that, for correct geometric delineation, any volume of variable cross‐section is ineligible for application of a single percentage contrast level. For spherical targets, the percentage contrast level required to yield correct delineation of the activated volume must be determined slice by slice. Proceeding in this manner yields the results presented in Fig. 5 for the small (5‐a), medium (5‐b), and large (5‐c) spheres.

Over the range of cross‐sectional areas presented by the three spherical targets, the required percentage contrast level is seen to vary greatly. The largest variation in cross‐sectional area occurs with the large sphere. Here, required percentage contrast levels decrease from a maximum in excess of 90% near the periphery of the sphere, where target areas are smallest, to a minimum of between 30% and 40% for the largest cross‐sections. The trend of larger percentage contrast levels associated with smaller cross‐sections proceeding to smaller percentage contrast levels associated with larger cross‐sections is clearly evident. This effect is a direct result of the decrease in contrast range associated with smaller target cross‐sectional areas, which in turn necessitates an increase in required percentage contrast levels.

#### *B.2 Sobel edge detection*

**Figure 6:**
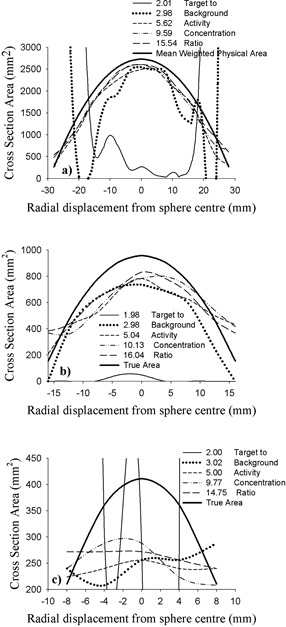
Cross‐sectional area delineated by the Sobel method as a function of radial distance from the center of the (a) large‐ (59‐mm), (b) medium‐ (35‐mm), and (c) small‐diameter (23‐mm) spherical targets for various target‐to‐background activity concentration ratios. The thick parabolic line in each image represents the mean weighted physical area of the sphere at each slice location.

Fig. 6 presents results obtained with the Sobel segmentation technique for the spheres. Experimental and mean weighted cross‐sectional areas are both plotted as a function of image distance from the center of each sphere.

Overall, the level of agreement achieved with this method is decidedly poor. In most cases, the disparity between measurement and reality increases as the TBAC ratio decreases. Most delineated cross‐sections are seen to fall short of the actual size, with the magnitude of the percentage deviation between the two values generally, but not exclusively, increasing for slice locations farther from the centers of the spheres.

At the lowest TBAC ratios (~2:1), this technique utterly fails in its application to the medium and large spheres. Cross‐sectional areas between 300% and 10,500% of the mean weighted values are indicated near the periphery of the large sphere for the two lowest TBAC ratios examined. These values are well off the plot scale of Fig. 6(a). Results are poorest for the small sphere, where even a cursory examination reveals the distinct discord between experimental measurements and the actual shape and size of the volume.

The best agreement between a Sobel‐derived cross‐sectional area and the corresponding mean weighted physical value occurs for the large sphere at TBAC levels of 5.6 and higher. However, the mean absolute percentage difference between measured and mean weighted cross‐sectional areas is 11.0% for a TBAC ratio of 5.6, 6.8% for a ratio of 9.6, and 6.5% for a ratio of 15.5.

#### *B.3 Watershed*

**Figure 7:**
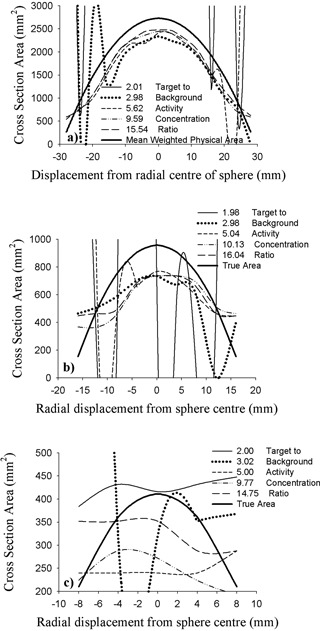
Cross‐sectional area delineated by the watershed method for the (a) large‐ (59‐mm), (b) medium‐ (35‐mm), and (c) small‐diameter (23‐mm) spheres for various target‐to‐background activity concentration ratios. The thick parabolic curve in each image corresponds to the mean weighted physical area at each slice location.

Fig. 7 presents results obtained with the watershed segmentation technique. Experimental and corresponding mean weighted cross‐sectional areas are again both plotted as a function of image distance from the center of each sphere.

A trend similar to that produced by the Sobel method is observed for the large and medium spheres, with most of the measured cross‐sections falling short of the mean weighted values. The greatest consistency between the watershed‐derived cross‐sectional areas and the corresponding mean weighted values occurs with the large sphere for TBAC levels of 9.6 and higher. The mean absolute percentage differences between watershed cross‐sections and the mean weighted physical values are 1.5% for a TBAC ratio of 9.6 and 5.1% for a ratio of 15.5.

For the large sphere, watershed results obtained with the lowest TBAC ratio (2.0) are opposite to those produced by the Sobel technique. In this case, the watershed cross‐sections at image locations nearer the center of the sphere are unrealistically large, ranging from 200% to 10,000% of true size. They are well off the plot scale of Fig. 7(a).

For the small sphere, the watershed method yields decidedly deplorable results that have no discernable trend with respect to TBAC ratio.

## V. DISCUSSION

Images consisting solely of a single uniform target fully encompassed by a homogeneous background should provide the simplest possible data set upon which to perform segmentation. The geometry of the experimental setup employed for the present investigation was specifically chosen to closely approximate this ideal case. Unfortunately, the low resolution characteristic of PET (on the order of 5 mm) gives rise to significant partial‐volume effects that severely hamper the segmentation of small objects. The problem of PET target volume delineation is further exacerbated by the presence of image noise.

The partial‐volume effects between target and background are modified by the finite wall thickness of the cylinders and spheres used in the present study. The walls produce a region devoid of activity that separates the hot target from the surrounding cool, less active background. Partial‐volume effects at wall locations produce voxels of three distinct compositions:

- Hot target activity, plus cold wall
- Hot target, plus cold wall, plus cool background
- Cold wall, plus cool background

The net result is threefold. First, the region of transition between target and background is enlarged. Second, the reconstructed activity in the transition region may drop below that of the background region. Third, the contrast between the target and the background can be suppressed.

Although an infinitesimal boundary might seem to be more desirable for a phantom study, the sharp interface that would be produced is in all likelihood a poor analog of the clinical situation, in which heterogeneous tumor and background activity uptake and the existence of microscopic tumor extensions serve to obfuscate the physiologic boundary between real biologic targets and their surroundings.

Standard radiotherapy practice is to encompass a gross target volume with a minimum margin of 5 mm to account for the possible presence of undetected subclinical involvement. Although the simple geometric arrangement employed for the present study cannot hope to fully reproduce the intricate complexities found in the clinical environment, the presence of inactive walls of finite thickness are not detrimental to clinical applicability.

Despite the inherent simplicity of the images used, delineation difficulties were encountered with each of the three segmentation approaches examined. Contrast‐based segmentation produces best results when cross‐sections are large—as evidenced by the manner in which the range of percentage contrast values that yield correct cross‐sections vary as a function of cylinder diameter. The range of percentage contrast values that produces agreeable results increases from a fairly narrow interval of about 6% for the large cylinder to approximately 25% for the smallest cylinder. This result is intuitively obvious, because the deleterious effects of image blur and noise are most significant for the resolution of small objects as opposed to large ones.

Also evident is the trend in the values of percentage contrast level required to yield correct cross‐sections—a trend that progresses from lowest with the largest cylinder to highest with the smallest cylinder. This result is also easily understood: large objects generally exhibit better low‐contrast resolution (contrast recovery) than do smaller ones.

Results obtained with the cylinders clearly indicate the ability of the threshold method to yield correct contour delineation, but they also reveal the liability of the approach. The percentage contrast level required to produce correct segmentation is clearly a function of target cross‐section size and hence requires at least some prior knowledge of the approximate physical extent of the object to be delineated. This necessity is clearly seen with the sphere results, where the percentage contrast levels required to correctly delineate individual cross‐sections range from less than 30% to more than 90%.

The more mathematically robust methodologies represented by Sobel edge detection and the watershed technique promise to eliminate the need to make estimates of target size before segmentation. However, both techniques produce unsatisfactory results when applied to the PET images examined here, despite the use of a simplified geometry. In addition, the enhanced activity concentration gradient across the target‐wall interface of the cylinders (due to finite wall thickness) would have been expected to confer a distinct advantage to these two segmentation approaches. However, results obtained from the cylinders reveal that both methods consistently identify cross‐sections that are smaller than those of the large cylinder and larger than those of the small cylinder. In both cases, the disagreement between experimental and physical cross‐sections increases as the contrast between target and background (TBAC ratio) decreases. Gradient‐based detection methods are well known to amplify the deleterious effects of image noise. Our observations strongly indicate the presence of substantial image noise in addition to partial‐volume effects.

For the large cylinder, the Sobel and watershed techniques both identify progressively smaller cross‐sections as target‐to‐background contrast decreases. For the small cylinder, the opposite is seen. Results obtained with the medium‐size cylinder clearly present a transitory state for the Sobel technique, in which all experimental cross‐sections are still smaller than the true physical size, but reductions in target‐to‐background contrast now result in the identification of cross‐sections of increased size. The relative agreement between experimental and physical cross‐sections seen with the Sobel technique for the medium‐size cylinder is thus the result of a fortuitous confluence of circumstances rather than an indication of encouraging results.

Results obtained with the spheres loosely exhibit the same trends observed with the cylinders. Larger cross sections are generally, but not exclusively, underestimated by both techniques. Similarly, the Sobel and watershed approaches generally both tend to overestimate smaller cross‐sections. The best results with the Sobel method are achieved with the large sphere and a TBAC ratio of 5.62 or higher. Thoroughly disappointing results are achieved with the small sphere over the entire range of TBAC ratios examined. The watershed approach leads to the same conclusions.

An examination of Fig. 6(a,b) reveals that, at the highest TBAC ratios, the Sobel technique underestimates the largest cross‐sections (central slice location) of the large and medium spheres, with their 1‐mm–thick walls, by 4.5% and 13.1% respectively. The large cylinder, with its thicker 4‐mm wall, has a diameter roughly equidistant between that of the medium and large spheres. For that cylinder, the Sobel technique identifies a cross‐section that is approximately 7% below its physical value at the higher TBAC ratios. As with the cylinder results, the sphere results strongly suggest that the image blur and noise inherent to PET are the primary sources of the difficulties experienced in the Sobel and watershed approaches to segmentation. A more definitive analysis of the effect of image blur and noise is beyond the scope of the present investigation.

## VI. CONCLUSION

Accurate geometric delineation of tumor volumes is essential if PET is to assume a significant quantitative role in both clinical diagnosis and radiotherapy management of cancer. Of the three segmentation techniques examined here, only the threshold method exhibited potential to faithfully reproduce cross‐sections of the simple geometric objects examined. Partial‐volume effects and image noise hobble the Sobel and watershed techniques. Because both of the latter methods fail to accurately segment simple geometries, they are unlikely to work on more complex clinical images.

Thresholding proved to be less susceptible to partial‐volume effects and image noise, but the dependence of the percentage contrast level on target size limits the usefulness of this technique. For the simple geometries provided by cylindrical volumes, the percentage contrast level required for correct segmentation ranges from about 30% to 65%. For the more realistic geometries provided by spherical volumes, slice‐specific percentage contrast levels ranging from less than 30% to more than 90% are required to yield correct cross‐sectional reproduction. Achieving the greatest conformity with physical reality would thus require the use of size‐dependent contrast levels, necessitating existing knowledge of the physical extent of the structures to be delineated. The unsatisfactory nature of such a requirement suggests contemplation of a single, suitably chosen percentage contrast level to be applied in all circumstances.

For clinical application, where avoiding underestimation of the full extent of tumor volume is desirable, the use of a lower percentage contrast level is indicated. Given the experimental data gathered from the three spherical targets, a contrast level of 28% would be appropriate for the PET scanner used here. This single percentage contrast level would yield inclusion of the minimum amount of surrounding background volume and at the same time ensure that no target cross‐section over the range presented by the study volumes is underrepresented. The less‐than‐satisfying nature of this conclusion is clear indication of the need for continued work with regard to the use of thresholding for PET target volume delineation if greater delineation accuracies are to be achieved.
